# Fano resonances induced by strong conductive coupling in cross-shaped metasurfaces for tunable EIT-like phenomena

**DOI:** 10.1038/s41598-024-69112-0

**Published:** 2024-08-09

**Authors:** Morteza Teymoori, Arda Deniz Yalcinkaya

**Affiliations:** 1 Institute of Biomedical Engineering, Boğaziçi University, Istanbul, Turkey; 2 Department of Electrical and Electronics Engineering, Boğaziçi University, Istanbul, Turkey; 3 Department of Microsystems Engineering (IMTEK), University of Freiburg, 79110, Freiburg im Breisgau, Germany

**Keywords:** Terhertz, Metamaterial, Cross-resonator, Fano, EIT, Conductive coupling, Metamaterials, Terahertz optics

## Abstract

Highly efficient Metamaterials are necessary for applications in sensing, communication, etc. Fano resonance and electromagnetically induced transparency-like phenomena are essential for obtaining high *Q*-factor and sensitive Metamaterials. Employing both numerical simulations and experimental analysis, we investigate the emergence of Fano resonance in cross-resonator Metamaterials facilitated by the conductive coupling between dark and bright resonators. We analyze the gradual shift of the fano resonance by tuning the dark resonator and finally form an electromagnetically induced transparency-like transmission peak. The strong coupling of the resonator is observed in the form of an anti-crossing and discussed through analytical models. We demonstrate that the coupling strength of the dark and bright resonance in our metamaterial is proportional to the asymmetry parameter, albeit at the cost of the Fano resonance’s *Q*-factor. The findings and methods introduced in this study can be used to develop highly efficient THz Metamaterials for various applications operable in room conditions.

## Introduction

The frequencies 0.1 to 30 THz have attracted tremendous interest during the recent decade. THz radiation has been explored in spectroscopy, imaging^1–3^, communication^4–6^, sensing, and detection of medical analytes with an ever-growing body of research^7,8^. The inherent characteristics of light-matter interaction in the THz range promote this growth of interest. However, light-modulation components are required for further implementation of THz-based technologies. In this regard, metamaterials attracted significant attention^7,8^. Metamaterials are intricate subwavelength structures that offer extraordinary behaviors determined by their design. This allows their customization based on the intended application. Metamaterials are predominantly resonant structures that confine energy in a narrow frequency band. Their performance is often measured by their quality factor ($Q-$factor), with a higher $Q-$factor indicating greater efficiency. Such efficiency indicates lower losses, higher sensitivity, and a more narrow-band response. Consequently, extensive research has been conducted to develop metamaterials with high Q-factors, as highlighted in several studies^9–13^.

There are two loss mechanisms in metamaterials, namely radiative and ohmic losses. The latter is rooted in electrical conduction, which can be minimized using high-conductivity materials like gold. Yet, mitigating these losses at higher frequencies and in smaller-scale structures becomes increasingly challenging, making radiation losses more significant^14,15^. These losses arising from re-emission or scattering of radiation are more complicated but are manageable through careful design. One such method involves leveraging the interference between two resonances, such as a dark and a bright resonance. Fano resonance, generally formed by breaking the symmetry of the resonator, effectively diminishes the radiative losses and enhances the $Q-$factor^15,16^. This principle has been applied in various designs across the literature to create more efficient metamaterials^17,18^. The distinct profile of Fano resonance, usually characterized by its asymmetric shape in the metamaterial’s response spectrum, can be fine-tuned to unveil additional phenomena. One such phenomenon is electromagnetically-induced transparency (EIT), typically observed in atomic systems. EIT manifests as a narrow linewidth transparency window in a rather wider opaque range of the spectrum, resulting from the destructive interference of two resonators with the same resonance frequency^11,19–21^. The optical and THz analogues of EIT system have been reported in the literature, showing potential in slow-light applications^22–25^. Furthermore, the strong interaction of the resonances at the EIT peak maximizes the field enhancement, offering greater sensitivity to the refractive index change in the vicinity of the resonator. This aspect is particularly promising for the development of sensitive optical devices and materials^26–28^.

In this study, we delve into the versatile functionalities of a seemingly straightforward but underappreciated resonator design, the Cross-Shaped resonator. Our focus is on exploring its tunability to exploit the advantages of Fano resonance and EIT-like phenomena. To explore this design and understand the behavior of each resonance, we employ both numerical full-wave simulations and experimental methodologies. With such a comprehensive approach, we aim to not only design a resonator with tuned properties but also contribute to the theoretical understanding and design steps by analyzing the resonance phenomena with analytical models.

## Results and discussion

**Figure 1:**
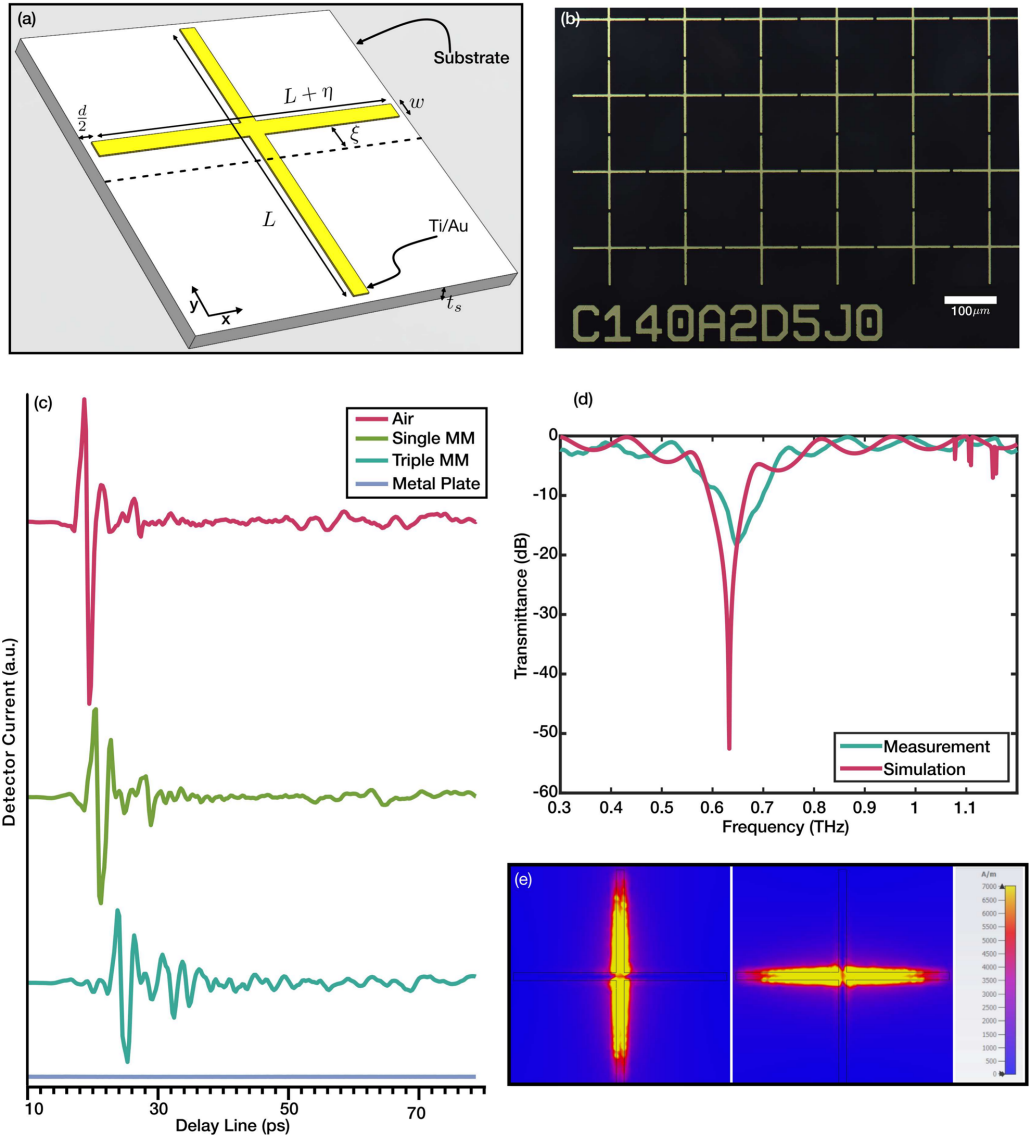
(**a**) The illustration of the resonator with critical dimensions. (**b**) Micrograph of the fabricated metamaterial with $L=140\,\mu$m, and $\xi =2\,\mu$m. (**c**) Initial Terahertz Time Domain Spectroscopy results with empty sample holder (air), single Metamaterial chip, three stacked metamaterial chips, and metal plate. (**d**) Comparison of transmittance spectra of the experimental measurement and simulation of a symmetrical metamaterial chip $(\xi =0)$. (**e**) The surface current densities of two excited dipole resonances ($f_0=$ 0.633 THz) by two perpendicular polarizations in the symmetric resonator.

The structure of the unit cell consists of two perpendicular arms with critical geometric dimensions illustrated in Fig. 1a. The incident THz radiation has a propagation vector in the *z*-axis while the polarization is either in the *x* or *y* axes. The latter will be called the asymmetry axis in the rest of the paper. The axis of asymmetry is assumed to be perpendicular to the off-center arm (Fig. 1a). The critical dimensions of the metamaterial that roughly controls the resonance frequencies were selected so that the resonances are in the frequencies with the most dynamic range. The arms’ initial length (*L*) (in the symmetric structure) was set to 140 $\mu$m, with both going through the center of the other one. The substrate thickness ($t_s$) is 500 $\mu$m. A metal width (*w*) of 5 $\mu$m is used for all the designs. The distance between resonators is fixed (*d*=35 $\mu$m) throughout the study. Thus, the periodicity of unit cells is constant in *y*-axis ($L+d$), but it varies in *x*-axis ($L+\eta +d$) as we study various $\eta$ values. The metamaterial chips were fabricated with $6\times 6$ mm$^2$ dimensions to avoid any beam-clipping. For each chip under study, measurements were conducted twice, accounting for both polarizations. As discussed in subsequent sections, chips with symmetric designs exhibit no sensitivity to polarization, meaning their performance is consistent regardless of the polarization orientation. However, for the asymmetric structures, the results depend significantly on the alignment of the polarization direction and axis of asymmetry (i.e. parallel or perpendicular). This distinction underscores the critical role of the structural design in affecting the optical behavior of these metamaterials.

The experimental measurements were carried out using a standard THz time-domain spectroscopy setup. To proceed with the measurements, the setup’s noise floor and dynamic range (DNR) were characterized using a 35 $\mu$m copper plate. Figure 1c shows that the transmission through a metal plate is zero. The calculated dynamic range justifies measurement between 0.4-1 THz with a minimum of 25 dB DNR (see supplementary Figure S1). The noise floor for the measurement range is calculated as 0.0322^29^. This substantiated the validity of the measurements in room conditions and relevance for a wide range of uses in THz applications for filtering, sensing, and detection.

The detected current for air, single metamaterial, triple-stacked metamaterial, and a metal plate is shown in Fig. 1c. The measurements were performed in room conditions without any vacuum or nitrogen purging, which is evident in the noise of the detector current. As is seen in the figure, the peak current is reduced in measurements with a metamaterial in the path of the beam. Additionally, the pulse is delayed to the detector when passed through the metamaterial. Such an effect is more evident when the beam passes through three stacked metamaterials. The presence of three different metamaterial chips on the path increased the delay and also the ripples after the main pulse due to the stronger and wider resonance (The detector current and frequency domain response of the individual and collective stacked metamaterial is illustrated in the Supplementary Information Figure S2).

A better understanding of the transmittance can be obtained from the results in the frequency domain. The transient current measured in the photoconductive antenna can be Fourier transformed into frequency domain amplitude and phase response. The transmitted signal through the sample can be written as

1 $$\begin{aligned} E_{(\omega )}=\textbf{E}_{(\omega )} e^{i \Phi _{(\omega )}} \end{aligned}$$

where $\textbf{E}_{(\omega )}$ and $\Phi _{(\omega )}$ are the frequency-dependent amplitude and phase. The final transmittance is calculated as the ratio of the transmitted signal through the sample and reference

2 $$\begin{aligned} T=\frac{\textbf{E}_{(\omega )} e^{i \Phi _{(\omega )}}}{\textbf{E}_{\textrm{ref} (\omega )} e^{i \Phi _{\textrm{ref} (\omega )}}}=\frac{\textbf{E}_{(\omega )}}{\textbf{E}_{\textrm{ref} (\omega )}} e^{i \Delta \Phi _{(\omega )}} \end{aligned}$$

where $\Delta \Phi _{(\omega )} = \Phi _{(\omega )} - \Phi _{\textrm{ref} (\omega )}$ is the phase delay. The measurement with nothing in the beam path (Air) was used as the reference. Figure 1d shows the experimental and computational spectral transmittance results for the symmetric resonator. A Finite Integration method was used for the simulations. A Quartz material model with $\varepsilon =$3.75 and loss $\delta =$0.0004 was used as the substrate. A lossless perfect electrical conductor model was used as the conducting layer to reduce computational costs while keeping the mesh quality high. As it is shown in the figure, the simulation and experiments match to a great extent with minimal variations. The simulation shows a resonance at 0.633 THz, which is 13 GHz lower than the measurement value. This variation results from the imperfections in the fabrication and possible differences between the substrate’s refractive index and the theoretical model used for the simulation. Additionally, the quality factor of the resonator is calculated as 6.89 and 4 for the simulation and experiment, respectively. This arises from the lack of ohmic losses in the simulations. The current density contours for both polarizations are shown in Fig. 1e. As the figure shows, the dipole resonance is only excited parallel to the incoming radiation’s polarization, and the perpendicular arm is completely dark. It must be noted that the dipole resonances for both polarizations happen at the same frequency as the lengths of the resonators are equal.

**Figure 2:**
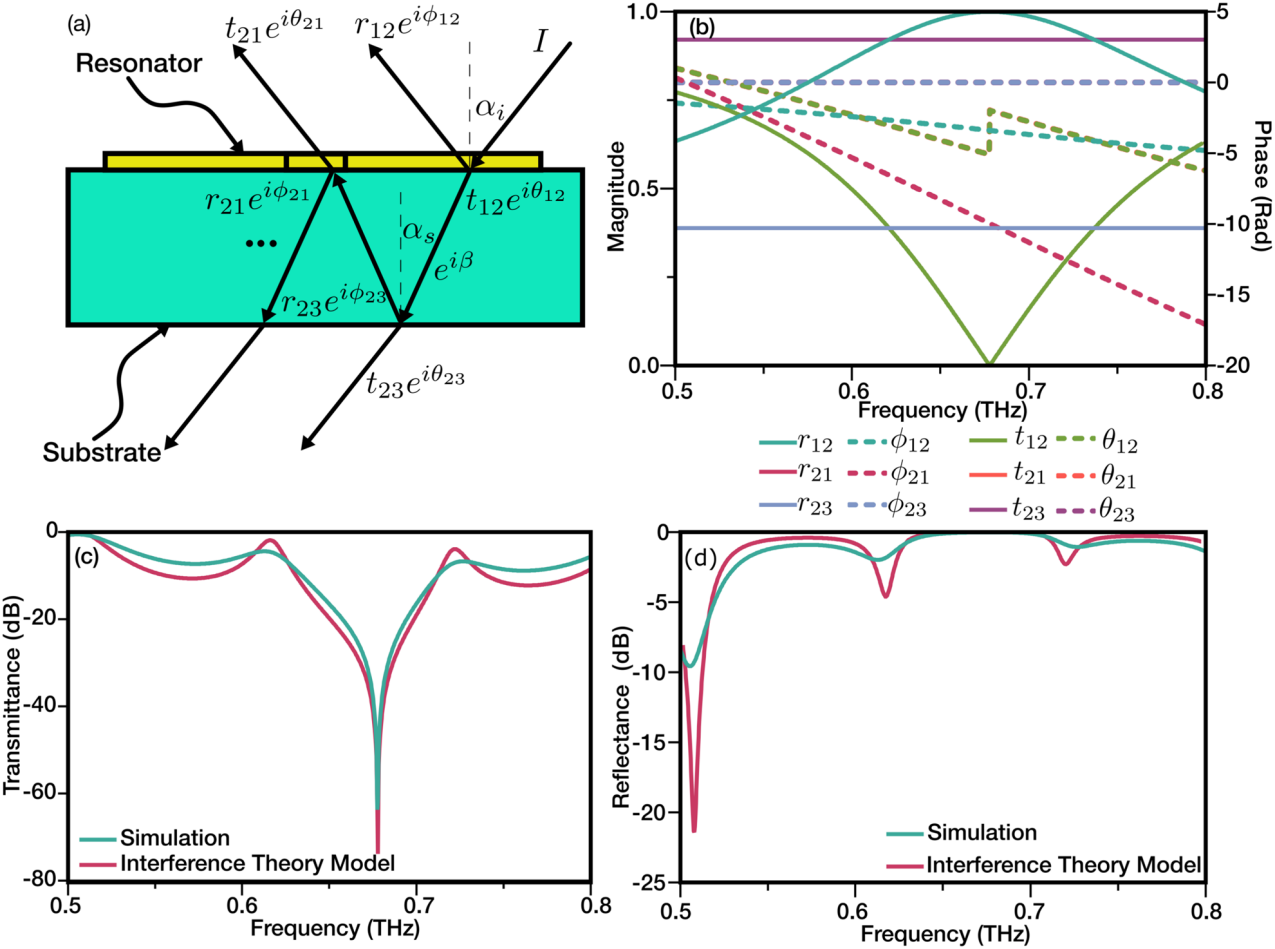
The interference theory model illustration (**a**) with the fitted transmittance and reflectance coefficient amplitude and phases (**b**). Note that $\theta _{23}$, $\phi _{23}$ and $\theta _{12}$, $\theta _{21}$ are overlapped. The calculated transmittance and reflectance of the interference model were compared to the results of the full-wave simulations (**c**,**d**).

It is also important to note in Fig. 1d that the wavy-like profile seen in the transmittance spectra of the metamaterial at frequencies outside the resonance region both in the simulations and experiments is rooted in the internal reflections due to the refractive index variation at interfaces. We used an interference model to verify this effect. Figure 2a illustrates the reflections and transmittance occurring in each interface. Having $\beta = -\sqrt{\varepsilon _g} \frac{k_0 t_s}{\cos (\alpha _s)}$, and $\alpha _s = \arcsin [\frac{\sin \alpha _i}{\sqrt{\varepsilon _g}}]$, the total reflectance and transmittance in the frequency domain can be derived as follows:

3 $$\begin{aligned} R = r_{12}\, e^{i \phi _{12}} \frac{t_{12} \,t_{21}\, r_{23} e^{ i(\theta _{12}+\theta _{21}+\phi _{23}+2 \beta )}}{1-r_{23}\, r_{21} e^{i(\phi _{23}+\phi _{21}+2 \beta )}} \end{aligned}$$

4 $$\begin{aligned} T = \frac{t_{12}\, t_{23} e^{i(\theta _{12}+\theta _{23}+\beta )}}{1-r_{23}\, r_{21} e^{i(\phi _{23}+\phi _{21}+2\beta )}} \end{aligned}$$

where $k_0$, $t_s$, and $\varepsilon _g$ are free space wavenumber, substrate thickness, and substrate permittivity, respectively. The reflectance and transmittance of the Quartz-air interface were calculated as:

5 $$\begin{aligned} r=\frac{n_i \cos \theta _t -n_t \cos \theta _i}{n_i \cos \theta _t + n_t \cos \theta _i} \end{aligned}$$

6 $$\begin{aligned} t=\frac{2n_i \cos \theta _i}{n_i \cos \theta _t +n_t \cos \theta _t} \end{aligned}$$

where $n_i$, $n_t$, $\theta _i$, and $\theta _t$ are the incident and transmit media refractive indices and angles, respectively. The rest was derived by eliminating the other components and adjusting the ports in the full-wave simulation. The calculated reflectance and transmittance amplitude and phases are illustrated in Fig. 2b. As expected, in the absence of the reflections from the Quartz-air interface on the back side of the substrate, the resonator’s transmittance ($t_{12}$) and reflectance ($r_{12}$) are smooth without any fluctuations around the resonance. The results of the interference theory model are shown in Fig. 2c,d, respectively. The figure shows that the full-wave simulations and the interference model match, thus verifying the source of the fluctuations in transmittance spectra. The quantitative mismatch in the dips and peaks is due to the limitations of the full-wave simulation, such as limited grid definition. To eliminate fluctuations around the resonance in transmittance spectra, we neglect internal reflection from the back surface of the substrate (Quartz-air interface) in our simulation models for the rest of the study. This was achieved by eliminating the vacuum gap between the quartz substrate and the second port and effectively matching the port with the quartz substrate. As a result, the waves inside the substrate that hit the Quartz-air interface are fully absorbed by the second port, preventing any reflections.

**Figure 3:**
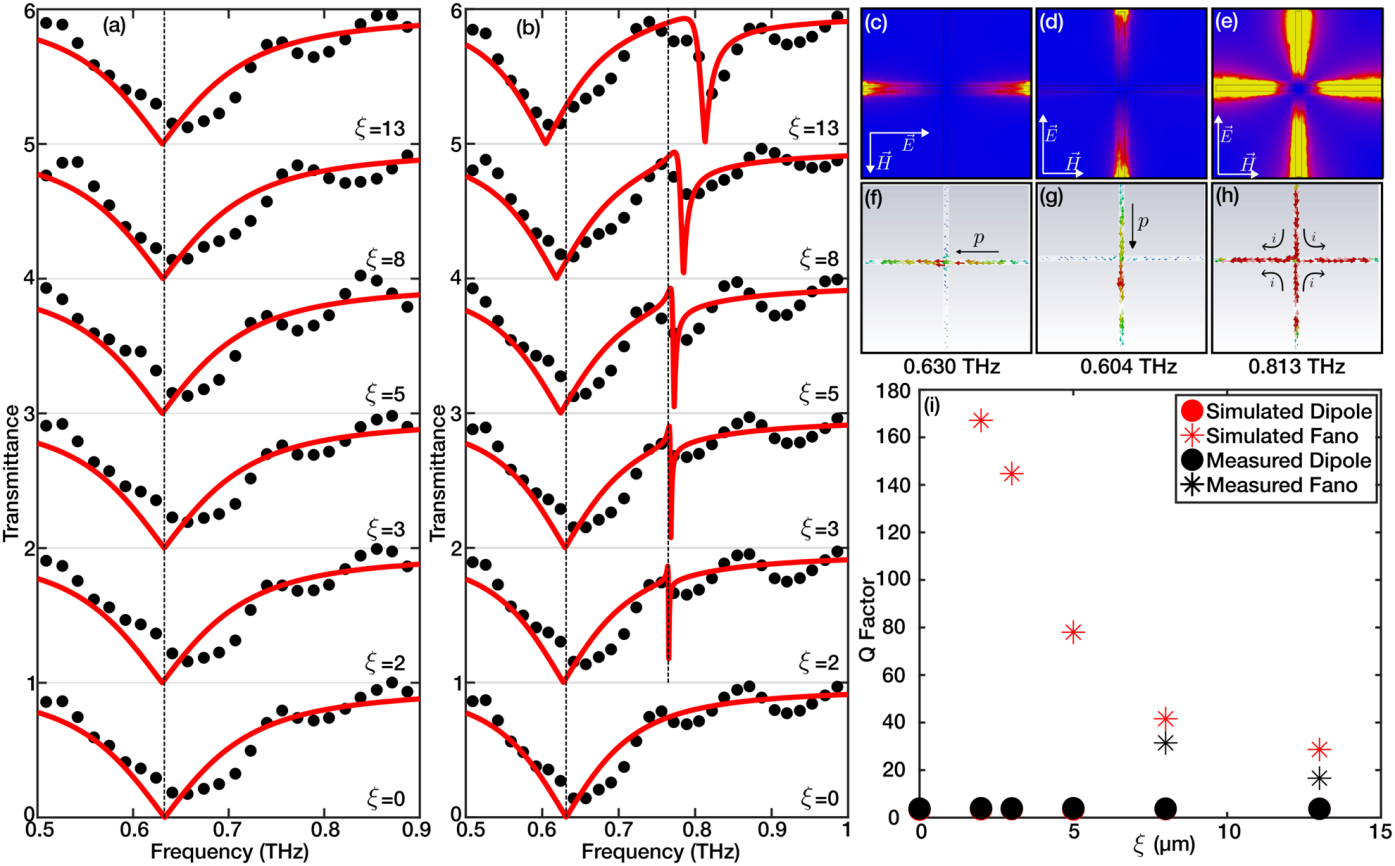
The transmittance spectra of different metasurfaces with various levels of asymmetry ($\xi$ = 0, 2, 3, 5, 8, and 13) for (**a**) perpendicular polarization and (**b**) parallel polarization to the axis of asymmetry. The E-field distribution and circulating surface current distribution in a resonator with $\xi$=13 for dipole resonance (0.630THz) perpendicular to the axis of asymmetry (**c** and **f**), dipole resonance (0.604THz) along the axis of asymmetry (**d** and **g**), and fano resonance (0.813THz) (**e** and **h**), respectively. The Q-factor for dipole and Fano resonances are calculated for both experimental and numerical results (**i**).

The symmetrical structure of the resonator yields a resonance dip with a low *Q*-factor invariant to polarization changes. However, the structure of the cross resonator offers a wide range of tunability. We will first explore the impact of asymmetrical adjustments in the positioning of the resonator’s arms relative to one another. Asymmetry is introduced by shifting the horizontal arm (aligned along the *x*-axis as shown in Fig. 1a) vertically along the *y*-axis (the axis of asymmetry), with the shift magnitude denoted by the parameter $\xi$ Fig. 1a. While the spectral transmittance response remains unchanged for polarization along the *x*-axis Fig. 3a, a distinct asymmetric resonance profile forms in measurements with polarization along the axis of asymmetry **Figure** 3b, hinting at its Fano-like nature. Figure 3c–h and show the E-field distribution and surface current density for Dipole and Fano resonances under perpendicular and parallel polarizations with respect to the asymmetry axis, respectively. Notably, the field enhancement at the Fano resonance point is significantly pronounced. Furthermore, the otherwise dark resonator (arm along the *x*-axis) that is not able to couple to the incoming wave is excited indirectly by coupling to the bright resonator (arm along the *y*-axis).

**Figure 4:**
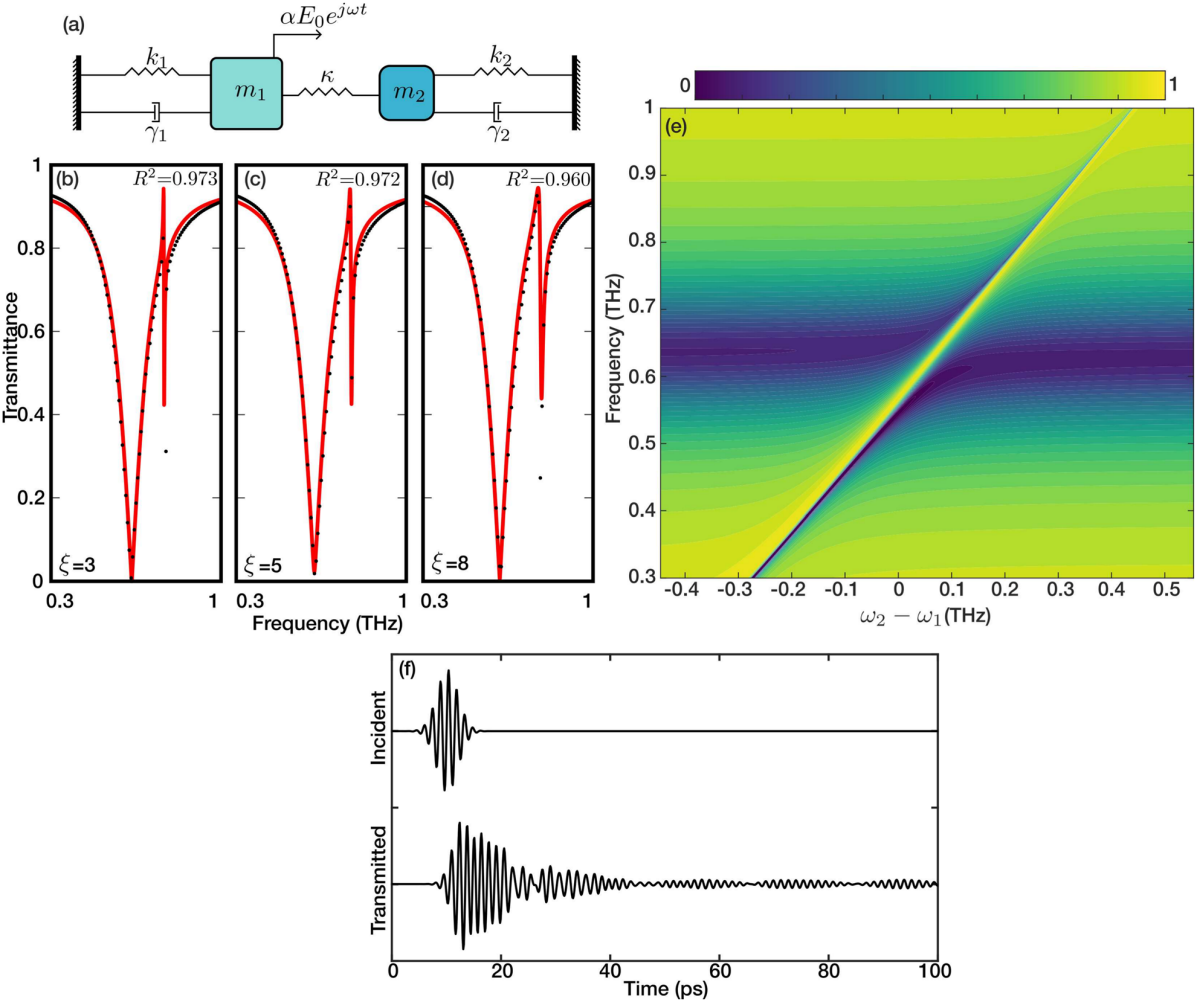
The coupled dark and bright resonators are represented with masses, springs, and dampers in a coupled oscillators model (**a**). The comparison of the full-wave simulation data (black dots) with the fitted analytical model (red lines) for three different values $(\xi =3, \xi =5$, and $\xi =8)$ of asymmetry (**b**–**d**). The contours of the transmittance spectra for $(\xi =8)$ across various dark mode frequencies show a strong anti-crossing associated with a strong coupling regime (**e**). The beating oscillation profile associated with the Rabi-splitting is also shown for the metamaterial with $(\xi =8, D=35$, and $\eta =50)$, which was calculated using a transient solver Full wave simulator (**f**).

The *Q*-factor is calculated as $\frac{f_0}{\Delta f}$, having ${\Delta f}$ as the 3-dB bandwidth, for dipole and fano resonances in simulation and experimental results and summarized in Figure 3**i**. It is observed that with increasing asymmetry (parameter $\xi$), the *Q*-factor for the secondary resonance dip diminishes, correlating with the characteristics of Fano resonances as documented in literature^30,31^. On the other hand, the *Q* factor of the dipole resonance does not experience any change as $\xi$ changes. Experimental results align well with *Q* factors of the dipole resonance with a slight variation. Nonetheless, the measured *Q*-factor for Fano resonances falls below simulation values, attributed to ohmic losses and the limited resolution of the THz-TDS setup. To validate the Fano nature of this asymmetric resonance and the above-mentioned shifts in resonance frequency, we used a coupled oscillators model illustrated in Fig. 4a. We assume that the vertical arm of the resonator (the arm along the direction of polarization) is the bright resonator ($m_1$). It can couple to the incoming wave (amplitude *E*) with a coupling coefficient ($\alpha$). In contrast, the perpendicular arm is a dark resonator ($m_2$) as it can only couple to the incoming light through the bright resonator. The coupling of resonators is modeled with a spring with coefficient $\kappa$. The resonance and loss behavior of the resonators is modeled with springs ($k_1$ and $k_2$) and dampers ($\gamma _1$ and $\gamma _2$). The equations of motion describing the state of the resonators and their coupling are:

7 $$\begin{aligned} m_{1} \ddot{x}_{1(t)} + \gamma _{1}\dot{x}_{1} + k_{1}x_{1} + \kappa (x_{1}-x_{2}) = \alpha \, E e^{j\omega t} \end{aligned}$$

8 $$\begin{aligned} m_{2} \ddot{x}_{2(t)} + \gamma _{2}\dot{x}_{2} + k_{2}x_{2} + \kappa (x_{2}-x_{1}) = 0 \end{aligned}$$

where $x_i$ ($i=1,2$) represents the amplitude of the oscillation in each resonator. Assuming that $m_2=A\cdot m_1$, we can transform equations 7 and 8 into their harmonic form as:

9 $$\begin{aligned} -\omega ^{2} X_{1} + j\omega \Gamma _{1} X_{1} + \omega _{1}^{2}X_{1} + \Omega ^2 (X_{1}-X_{2}) = \zeta E \end{aligned}$$

10 $$\begin{aligned} -\omega ^{2} X_{2} + j\omega \Gamma _{2} X_{2} + \omega _{2}^{2}X_{2} + A\,\Omega ^{2} (X_{2}-X_{1}) = 0 \end{aligned}$$

where $\Omega = \kappa /m_1$, $\zeta = \alpha /m_1$, $\Gamma _i = \gamma _i / m_i$, and $\omega _i$ ($i=1,2$) are the mass-normalized coupling strength between oscillators, coupling strength of the bright oscillator to the incoming wave, damping coefficients, and resonance frequency of each oscillator, respectively. The formulation for $X_{1}$ and $X_{2}$ can be determined by converting the above system of equations into matrix form.

11 $$\begin{aligned} \begin{bmatrix} -\omega ^{2}+j\omega \Gamma _{1} + \omega _{1}^{2} + \Omega ^{2} &{} -\Omega ^{2} \\ -A\Omega ^{2} &{} -\omega ^{2}+j\omega \Gamma _{2} + \omega _{2}^{2} +A\Omega ^2 \end{bmatrix} \begin{bmatrix} X_1 \\ X_2 \end{bmatrix}=\begin{bmatrix} \zeta E\\ 0 \end{bmatrix} \end{aligned}$$

Solving for $X_{1}$ and $X_2$ give the following:

12 $$\begin{aligned} X_{1}= \frac{-\omega ^{2} + j\omega \Gamma _{2} + \omega _{2}^{2} + A\,\Omega ^{2}}{ (\omega ^{2}-j\omega \Gamma _{1} - \omega _{1}^{2} - \Omega ^{2})(\omega ^{2}-j\omega \Gamma _{2} - \omega _{2}^{2} - A\Omega ^{2})-A\Omega ^{4}}\,\zeta E \end{aligned}$$

13 $$\begin{aligned} X_{2}= \frac{A\Omega ^2}{ (\omega ^{2}-j\omega \Gamma _{1} - \omega _{1}^{2} - \Omega ^{2})(\omega ^{2}-j\omega \Gamma _{2} - \omega _{2}^{2} - A\Omega ^{2})-A\Omega ^{4}}\,\zeta E \end{aligned}$$

Assuming an incidence field with a unit amplitude, the transmittance of the coupled system can be obtained via $T \triangleq 1+\alpha X_{1i}$, where $X_{1i}$ is the imaginary part of the $X_1$.

The analytical model was fitted to the full-wave simulation results for three different metamaterials with various asymmetry parameters ($\xi =3$, 5, and 8). As seen in Fig. 4b-d, the analytical model is a good fit for the simulation data. A closer look at the fitting parameters, briefly $\gamma _1=1.97\times 10^{11}$, $\gamma _2=10^{7}$, $\Omega _{(\xi =3)}=3.3\times 10^{11}$, $\Omega _{(\xi =5)}=3.65\times 10^{11}$, and $\Omega _{(\xi =8)}=4.65\times 10^{11}$  rad/s, shows that the oscillator 1 is significantly lossier (low-*Q*) than oscillator 2 (high-*Q*), which matches with the mechanism of the fano resonance. Additionally, comparing the losses with coupling between the oscillators ($\Omega > \Gamma _1 + \Gamma _2$), the coupling strength is significantly higher than the combination of the linewidth of both resonances at all asymmetries. This illustrates that the coupled system operates in a strong coupling regime^32,33^. Moreover, using the same parameters from the fitted model ($\xi$=8), the spectra of multiple cases with detuned dark resonators were calculated and illustrated with a contour map in Fig. 4e. As illustrated in the figure, the strong coupling of resonances is also manifested as anti-crossing behavior analogous to the Rabi-Splitting phenomena in atomic systems. The calculated transient oscillations using a Time-Domain solver in Full-wave simulation software confirm the analogy of rabi-splitting for the metamaterial with asymmetry parameter $\xi =8$ Figure 4f. The transmitted wave’s beating profile indicates the strong energy oscillation between two oscillators as well^34^. Going back to the observations in Fig. 3a,b, it is seen that as the asymmetry parameter increases, the dipole resonance perpendicular to the asymmetry axis remains the same, whereas for the parallel polarization measurements, the dipole and Fano resonances diverge further (with the dipole resonance experiencing a redshift and the Fano resonance a blueshift). Comparing the results with the coupled oscillator models, it is evident that such observations are associated with increased coupling strength between the bright and dark resonances.

**Figure 5:**
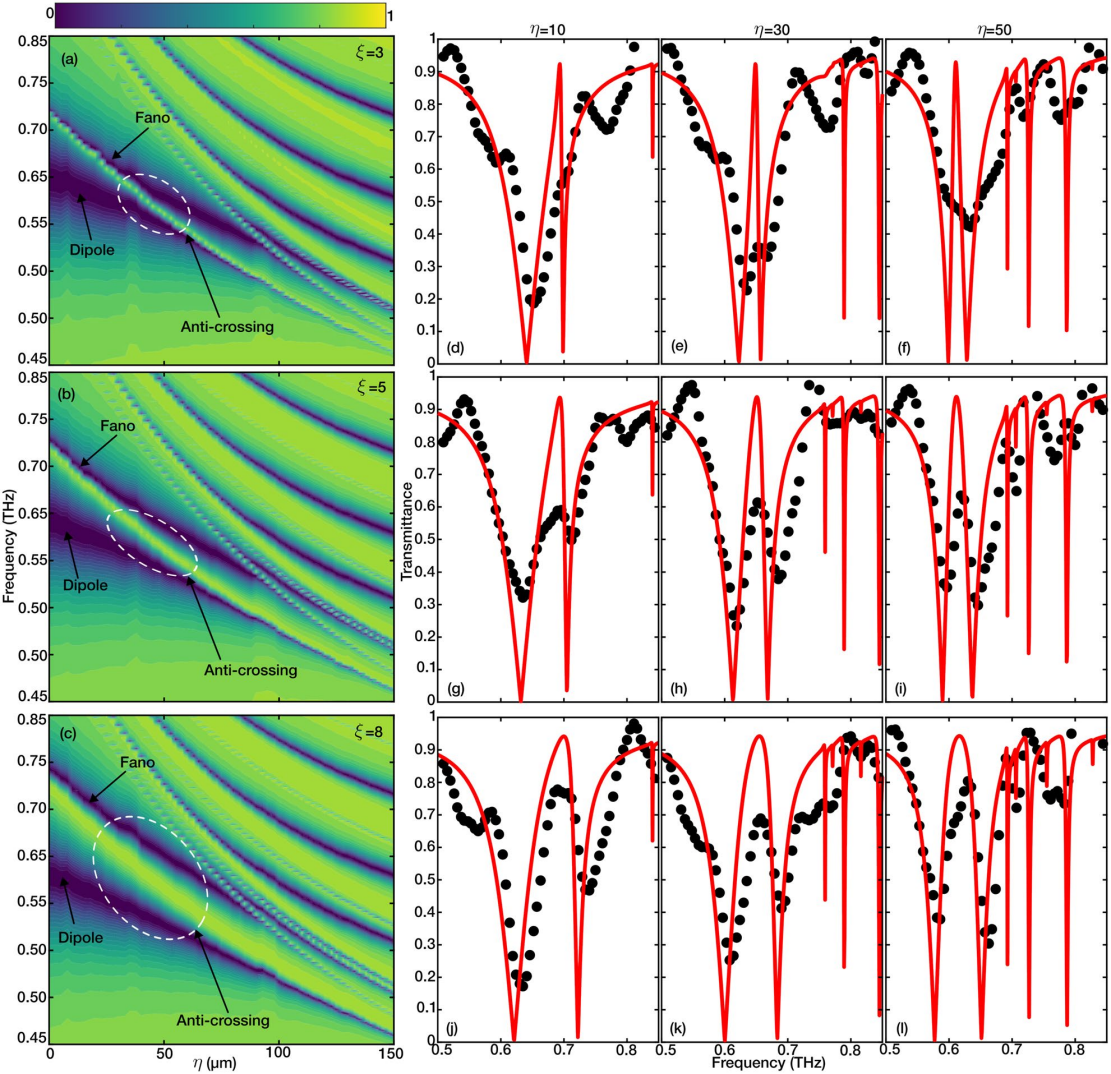
The evolution of the dipole and fano resonances are displayed in the form of a heat-maps for $\xi =3$, $\xi =5$, and $\xi =8$ in (**a**–**c**), respectively. The tunability of the fano and formation of a transparency window is demonstrated with three $\eta$ values (10, 30, and 50) for three $\xi =3$, $\xi =5$, and $\xi =8$ (**d**–**l**). The black dots and the red continuous lines represent the experimental and simulation results, respectively.

The anti-crossing illustrated in Fig. 4e offers a transparency window with a high *Q* factor. The EIT-like behavior is formed when the resonance frequencies of the dark and bright resonators are approximately equal^16^. Thus, in order to form the EIT-like behavior in this metamaterial, we modify the dark resonance by changing the length of the arm perpendicular to the axis of asymmetry. The length of the dark resonator is adjusted using the parameter $\eta$. As $\eta$ increases, the frequency of the Fano resonance moves towards the dipole resonance. Figure 5a–c show the shifts in the frequencies of the dipole and Fano resonances with regard to the change in $\eta$. The dipole and Fano resonance bands are indicated in the figures. As $\eta$ increases, the dipole resonance band tightens while the Fano resonance widens. The two resonances exhibit an anti-crossing, marked with a white dashed circle. The width of the anti-crossing, associated with the coupling strength, is different for various values of asymmetry parameters, indicating stronger coupling for higher asymmetry values. The transparency window has the highest quality factor at the anti-crossing location, which decreases as the $\xi$ increases. This is also evident in the transmittance profile of the metamaterials with different $\xi$ and $\eta$ values illustrated in Figure 5d–l. As seen in the figure, the experiments match the simulation very well, except for the very high Q resonances, where the THz-TDS setup’s resolution is insufficient. It must be noted that the narrow resonance bands illustrated in the simulation results of Figure 5 at higher frequencies than the resonators’ are attributed to the lattice modes. Such lattice modes form as the light scatters from an array of sub-wavelength structures and are inversely proportional to the periodicity parameter of the array^35^. As seen in Fig. 5a–c, as $\eta$ increases, the periodic parameter of the unit cell increases, thus the lattice mode frequency reduces.

**Figure 6:**
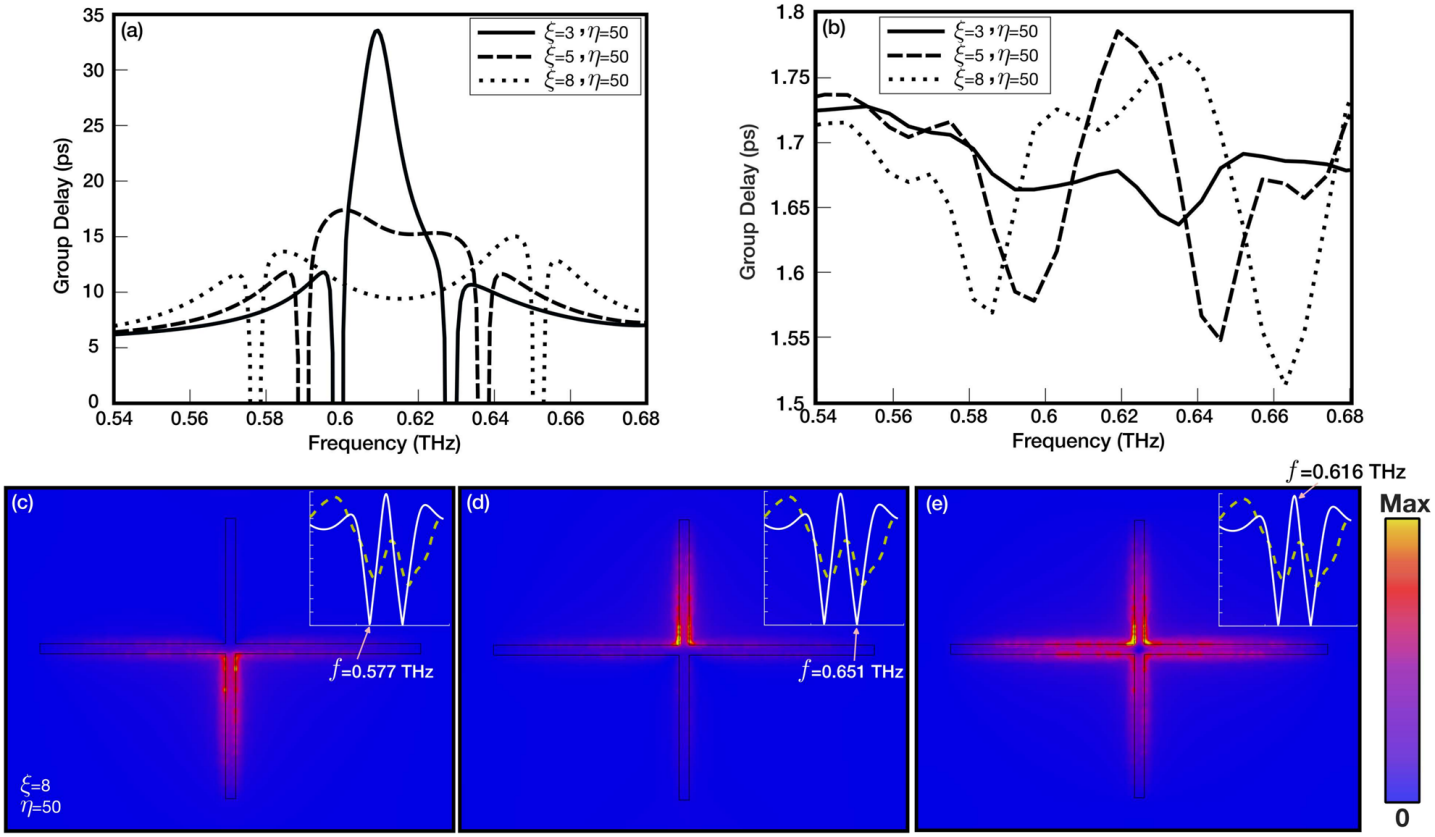
Group delay profile of metasurfaces with $\eta$=50$\mu$m and $\xi$= 3, 5, and 8$\mu$m were calculated from simulation (**a**) and experimental (**b**) results. The surface current density for a metasurface with $\eta$=50$\mu$m and $\xi$=8 $\mu$m at first (0.577THz) (**c**) and second (0.651THz) (**d**) resonance dips compared to the EIT peak (0.616THz) (**e**).

The numerical and experimental group delay ($\frac{ d\omega }{d\phi }$) is calculated for metasurfaces with $\eta$=50 and $\xi$=3,5, and 8 are shown in Fig. 6a and 6b, respectively. As it is seen, the slow light characteristic is most pronounced in the model with $\xi$=3 in simulations. As the asymmetric parameter increases, group delay at the EIT point decreases. This is expected as the coupling becomes stronger with asymmetry, the slow-light character diminishes^16^. Although the experimental group delay profile qualitatively matches the simulations, the pronounced profile seen in simulations for the model with $\xi$=3 cannot be seen due to the low resolution of the measurement method. Besides slow-light applications, EIT peaks can be useful for sensing due to their higher field enhancement around the resonator, as shown in Fig. 6c–e for the model with $\xi$=8 and $\eta$=50. The figure illustrates that while the first and second resonance dips exhibit partial field concentration in the resonator’s lower or upper arm, the EIT peak displays the highest field concentration values in all four arms of the resonator. Additionally, compared to the resonance dips or dipole resonance Fig. 1e, an increased surface area of the excited fields suggests that EIT peaks might find applications in sensing and detection by potentially increasing the light-matter interaction.

## Conclusion

In this study, we explored the overlooked capabilities of a cross-resonator, uncovering its potential for achieving high-*Q* Fano resonances and examined the resonance mechanisms through analytical techniques. Leveraging the design versatility of the cross-resonator, we fine-tuned the Fano resonance to create transparency windows analogues of Electromagnetically Induced Transparency (EIT) observed in atomic systems. Our design was rigorously modeled numerically, confirmed through experimental validation, and the underlying principles and mechanisms of the metamaterial were thoroughly analyzed with coupled oscillators model. We found that increasing the asymmetry parameter enhances the interaction between the bright and dark resonators, albeit at the cost of a reduced *Q*-factor for the Fano resonance. The resultant high-*Q* Fano and EIT phenomena facilitate significant field enhancement, opening avenues for the development of THz sensors and slow-light applications. Notably, discrepancies in resonance intensity between experimental outcomes and simulation predictions were attributed to the limited resolution of the THz-TDS equipment and the ambient conditions under which experiments were conducted, suggesting the feasibility of applications that are less demanding.

## Methods

The metamaterials were fabricated using the lithographic definition with AZ5214E photoresist. A physical vapor deposition (e-beam evaporation), followed by a lift-off process, was used to form resonators with Titanium/Gold metal films (5 nm/135 nm). An optical microscope image of a sample fabricated metamaterial is shown in Fig. 1b.

The THz-TDS setup consists of a femtosecond laser with a central wavelength of 1560 nm which generates a series of repetitive pulses. A beam splitter is used to divide the laser beam where one of the beams is focused on a low-temperature (LT) grown InGaAs/InAlAs photoconductive antenna. The emitted THz radiation is focused on the sample, and the transmitted THz radiation is then detected and measured by a LT InGaAs/InAlAs photoconductive detector. The detector is gated by the second laser beam, the time delay of which is controlled and swept using a mechanical delay line. The photocurrent measured using the lock-in-amplifier is reported as a function of the time delay introduced by the mechanical delay line.

### Supplementary Information

Supplementary Information.

## Data Availability

Data sets generated during the current study are available from the corresponding author upon reasonable request.
