# Supplementary material for: Fano resonances induced by strong conductive coupling in cross-shaped metasurfaces for tunable EIT-like phenomena
Source: Sci Rep. 2024 Aug 9;14:18556. doi: 10.1038/s41598-024-69112-0 (PMC11316014; doi:10.1038/s41598-024-69112-0)
Supplement: Supplementary file 1 — Supplementary Information. [file 41598_2024_69112_MOESM1_ESM.pdf]

Fano Resonances Induced by Strong Conductive Coupling in Cross- Shaped Metasurfaces for Tunable EIT-like Phenomena

Morteza Teymoori*, Arda Deniz Yalçınkaya

Supplementary Figures

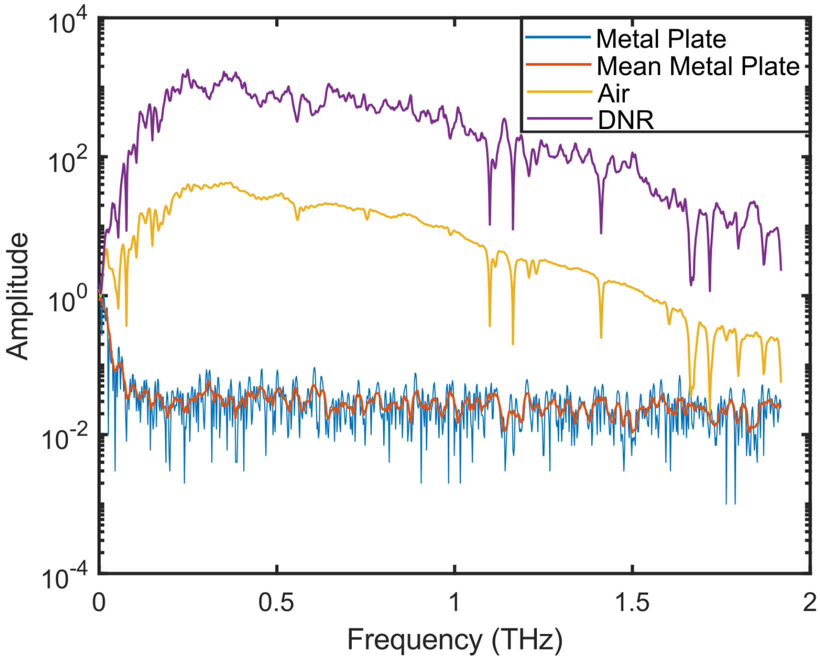

Figure 1: The Fourier transform of the detected signal with a metal plate in the beam path (noise floor), mean noise, and calculated dynamic range (DNR).

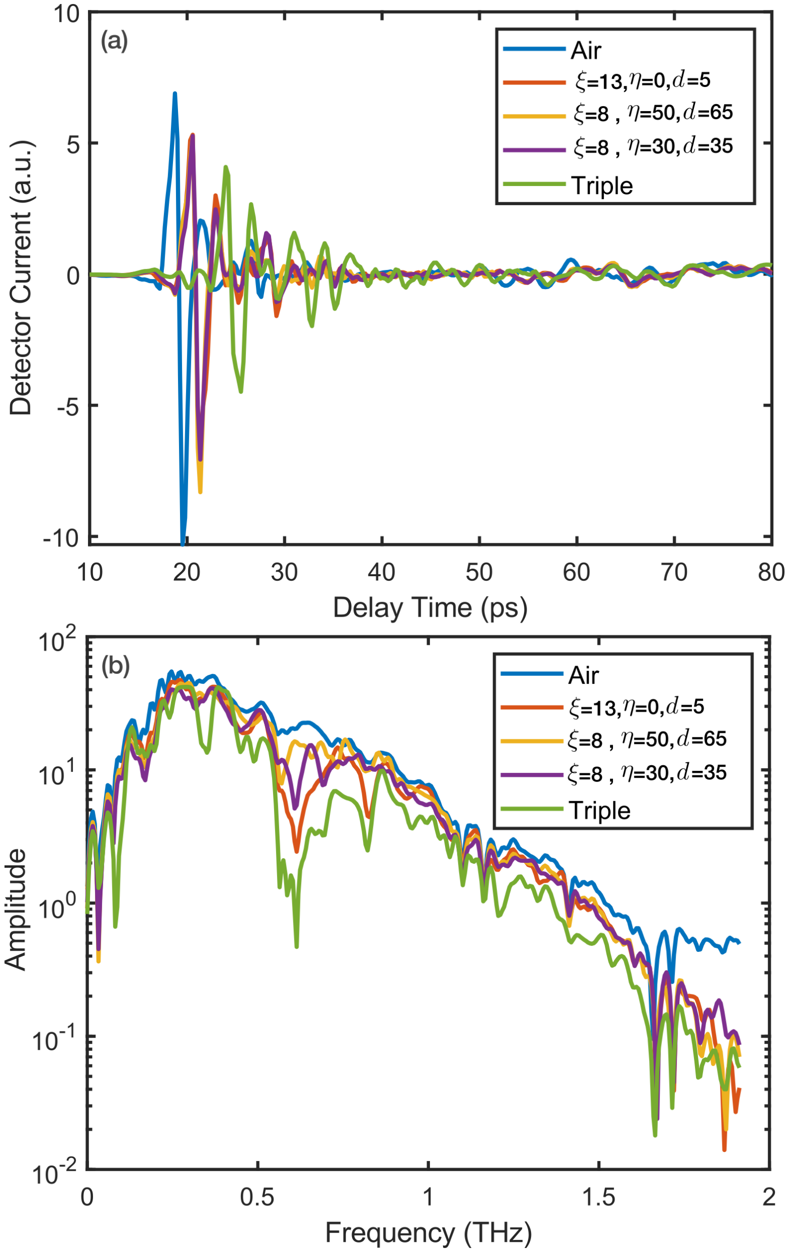

Figure 2: (a) Raw measurements of photocurrents from the air, metasurfaces with various geometrical parameters, and their stacked combinations. (b) Fourier transform of these photocurrent measurements.
